# Creep in nitroimidazole inhibitory concentration among the *Entamoeba histolytica* isolates causing amoebic liver abscess and screening of andrographolide as a repurposing drug

**DOI:** 10.1038/s41598-023-39382-1

**Published:** 2023-07-27

**Authors:** Aradhana Singh, Tuhina Banerjee, Sunit Kumar Shukla, Soumya Upadhyay, Ashish Verma

**Affiliations:** 1 Department of Microbiology, Institute of Medical Sciences, Banaras Hindu University, Varanasi, 221005 India; 2 Department of Gastroenterology, Institute of Medical Sciences, Banaras Hindu University, Varanasi, 221005 India; 3 Department of Life Sciences, Banasthali Vidyapeeth, Jaipur, 302001 India; 4 Department of Radiodiagnosis and Imaging, Institute of Medical Sciences, Banaras Hindu University, Varanasi, 221005 India

**Keywords:** Clinical microbiology, Parasitology, Gastroenterology

## Abstract

Infections by *Entamoeba histolytica* (*E. histolytica*) lead to considerable morbidity and mortality worldwide and treatment is reliant on a single class of drugs, nitroimidazoles. Treatment failures and intermittent reports of relapse from different parts of world indicate towards development of clinical drug resistance. In the present study, susceptibility testing of clinical isolates of *E. histolytica* was carried against metronidazole and tinidazole. Additionally, anti-amoebic property of active compounds of *Andrographis paniculata* was also evaluated. Prevalence of metronidazole resistance gene (*nim*) in patients attending hospital was also done to get comprehensive insight of present situation of drug resistance in *E. histolytica.* Mean inhibitory concentration 50 (IC50) value of *E. histolytica* isolates against metronidazole and tinidazole was 20.01 and 16.1 µM respectively. Andrographolide showed minimum mean IC50 value (3.06 µM). Significant percentage inhibition of *E. histolytica* isolates by andrographolide was seen as compared to metronidazole (*p* = 0.0495). None of *E. histolytica* isolates showed presence of *nim* gene. However, in stool samples from hospital attending population, prevalence of *nim*E gene was found to be 76.6% (69/90) and 62.2% (56/90) in diarrheal and non-diarrheal samples respectively. Inhibitory concentration of commonly used nitroimidazoles against clinical isolates of *E. histolytica* are on rise. Percentage inhibition of *E. histolytica* isolates by andrographolide was significantly higher than control drug metronidazole.

## Introduction

The choice of drugs for the treatment of infections by *Entamoeba* usually depends on the diagnosis and the severity of the disease. The World Health Organization (WHO) recommends that all patients infected with *Entamoeba histolytica* (*E. histolytica*) should be treated^1^. However, owing to the unavailability and the associated toxicity, treatment of infections due to *E. histolytica* is reliant on a single class of drugs, nitroimidazoles, for more than half a century^2^. The safety and efficacy of the nitroimidazoles in all forms of amoebiasis has been firmly established^3^. The most used agent from this class is metronidazole. Metronidazole has been the front-line choice for a number of anaerobic and protozoan infections worldwide.

However, indiscriminate overuse, over-the-counter (OTC) sales and inappropriate treatment regimens has resulted in the increase in the minimum inhibitory concentration (MIC) of the drug^4^. Appearance of the metronidazole resistant strains *in-vivo* and *in-vitro* can be an indication of the emerging drug resistance. However, the studies about the metronidazole resistance and the associated mechanisms from India are very limited^5^. Detection of resistance genes from the clinical samples without pure isolates can be helpful in epidemiological studies on the distribution of the resistance genes. Additionally, the antimicrobial susceptibility testing through culture methods provides information of the resistance genes of only micro-organisms which are capable of growing under given conditions.

Cases of treatment failures even after adequate therapy from different parts of the world indicate development of clinical drug resistance. Reports of recurrence in the cases of extraintestinal manifestation of *E. histolytica* are on rise^6–9^. There are reports of multiple relapse and treatment failures despite following appropriate management^6^. Nonetheless, resistance to metronidazole, although reported in *Giardia lamblia*, *Trichomonas vaginalis* and *Leishmania donovani* is still not documented in the case of *E. histolytica* isolates^10–13^.

Ten nitroimidazole resistance genes (*nim A* to *nim J*) have been identified till date that confer reduced sensitivity to 5-nitroimidazole drugs^14–16^. The proposed mechanism for the resistance by *nim* gene states that they encode an enzyme called, 5-nitroimidazole reductase, that inhibit the formation of the nitroso radicals critical for the antimicrobial activity^17^. It has been demonstrated that the high level of metronidazole resistance can be easily induced in strains containing *nim* gene^15^. The increased frequency of the *nim* gene even after short exposure to metronidazole treatment has been reported.

Since last 60 years, no new drug has developed against *E. histolytica* infections, despite the urgencies set by National Institute of Allergy and Infectious diseases (NIAID) for drug development for treatment of category B pathogens. *In-vitro* drug resistance in metronidazole is not a serious problem presently, however, the increasing inhibitory concentrations and cases of treatment failures is heralding towards the clinical resistance^6–8^. Additionally, there are several adverse effects associated with the long-term use of metronidazole including diarrhoea, loss of appetite, metallic flavour, induction of genotoxic effects, DNA damage as well as oxidative cell damage^3,18,19^. The most critical reactions include effect on central nervous system, peripheral neuropathy, ataxia, vertigo, convulsions and cerebellar toxicity^20–22^. Thus, it becomes important to reconsider the risk benefit relation and individual’s susceptibility in case of metronidazole prescription.

The revaluation of different medicinal plants for their therapeutic properties has gained much importance due to the emerging drug resistance to the commonly used agents. One such plant species is *Andrographis paniculata* (*A. paniculata*). *A. paniculata* is a medicinal herb with numerous pharmacological properties. The abundant therapeutic use of this plant has been demonstrated in the tropical and sub-tropical regions of South-east Asia^23^. These regions are also endemic for the *Entamoeba* infections leading to considerable morbidity and mortality^24–26^. Andrographolide is the sole major compound isolated from *A. paniculata*, which has been studied for numerous medicinal properties. Neo-andrographolide and andrograpanin are the other dominant and important diterpenoids isolated from the aerial part of *A. paniculata*^27^*.* In our experience, significant anthelmintic property, and the quantitative estimation of the marker compounds in the extracts of leaves of *A. paniculata* has been recently reported against human hookworm isolates^28^.

In our tertiary care center, we have previously studied high prevalence of *E. histolytica* in amoebic liver abscess (ALA) and high recurrence of ALA in a 2 year follow up study^8,29^. The location of the present study is on the Ganga basin which has been associated with higher incidence of ALA in an endemic neighbouring country. The unusual rate of recurrence could hint towards the gradual development of the clinical resistance towards the commonly used drugs in this geographical area. Consequently, we hypothesized whether clinical resistance with metronidazole treatment is reflected in drug susceptibility testing of *E. histolytica*. With this background, the objective of this study was to evaluate the drug susceptibility of the clinical isolates of *E. histolytica* causing ALA against metronidazole and tinidazole. Along with it, the analysis of the anti-amoebic activity of active compounds of *A. paniculata* (andrographolide, neo-andrographolide and andrograpanin) was done. Additionally owing to the dearth in existing data, the prevalence of metronidazole resistance gene (*nim* gene) in stool samples of patients attending hospital was also carried out, to provide a comprehensive picture of the current situation of drug resistance in *E. histolytica.*

## Results

### Confirmation of the samples and demographic details

**Figure 1:**
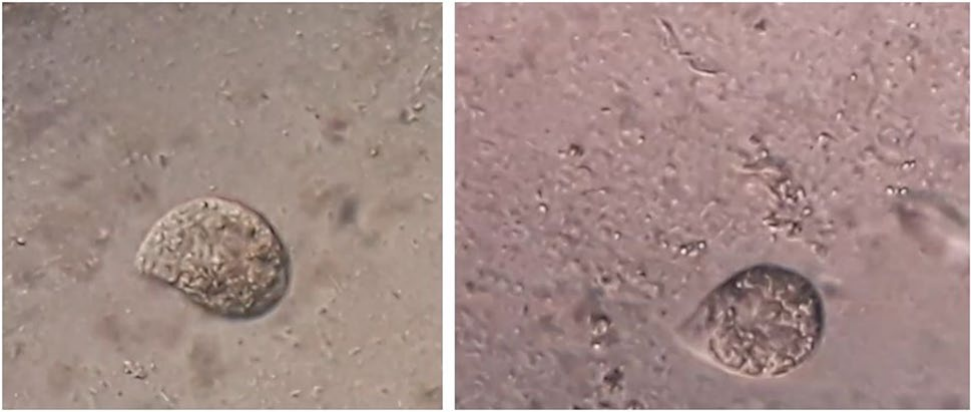
Microscopic image of the trophozoites in the liver aspirate samples (40x).

All the 15 liver aspirates included in the study were found to be positive for *E. histolytica* through nested multipex polymerase chain reaction (PCR) for *Entamoeba* sp. However, only 2 (13.3%) stool samples of these ALA patients were positive for *E. histolytica*. As we have previously reported increased recurrence rate in ALA patients thus, the isolation of the trophozoites were carried out from the liver aspirate samples. Figure 1 shows the presence of the trophozoites in the liver aspirate samples. Among the diarrheal samples included in the present study 3 (3.3%) samples were positive for *E. histolytica*.

The mean age of the ALA patients was 41.06 ± 7.3 years. Majority (13/15, 86.6%) of the participants were males. Considering their residence, 9 (60%) participants were from rural background and 6 (40%) were from urban areas. Out of the total, 5 (33.3%) participants had received higher education (above class 5th), 7 (46.6%) participants had primary education and 3 (20%) were uneducated. Majority (11/15, 73.3%) participants were employed. Five (33.3%) participants were from low and 10 (66.6%) participants were from middle economic status. In the present study, the incidence of ALA was more frequent in middle-aged males with rural background. However, no significant correlation was found between the demographic factors and the incidence of the ALA owing to the inclusion of only the diseased ALA cases (no controls) and the small sample size of the study.

### Drug susceptibility testing of *E. histolytica* clinical isolates

**Figure 2:**
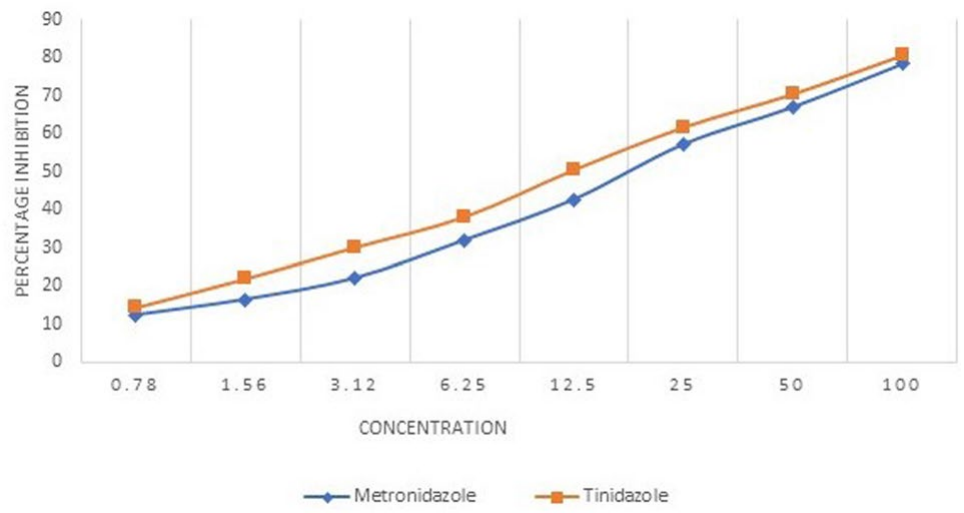
Percentage inhibition of *E. histolytica* clinical isolates by metronidazole and tinidazole.

The mean IC50 value of the *E. histolytica* isolates against the metronidazole and tinidazole was found to be 20.01 µM and 16.1 µM respectively. The mean IC50 values were significantly higher (*p* = 0.022) for metronidazole as compared to the tinidazole. Figure 2 shows the graphical representation of the mean percentage inhibition of the 15 clinical isolates of *E. histolytica* by the metronidazole and tinidazole. As it is evident from the Fig. 2 that the inhibition percentage of the tinidazole was higher than the metronidazole against the clinical isolates of *E. histolytica* throughout the concentration range included in the study. A list of studies showing details of the drug susceptibility testing of the *E. histolytica* isolates using various assays has been shown in Table 1.

**Table 1:** List of studies showing details of the drug susceptibility testing of the *E. histolytica* isolates using various assays.

Species	Isolate type	No	Culture type	Study site	Method used	Detection of antiamoebic activity	Concentration	Reference
E. h (HK-9)	Standard strain	1	Axenic	Missouri U.S	Micro dilution plate method	Ability to inhibit the incorporation of thymidine and parasite replication	ED50- Metronidazole:0.17–0.35 µg/ml Dihydroemetine: 0.15 µg/ml Emetine:1.96–2.83 µg/ml Chloroquine:0.56–1.57 µg/ml Paromomycin: 48.1–60.7 µg/ml	^59^
E. h	Clinical isolates	30	Axenic	Bangkok, Thailand	Tube method	Quantitative assessment using neutral red stain	MIC- Metronidazole: 0.0625–0.125 µg/ml Dihydroemetine: 0.125–1 µg/ml Tinidazole: 0.0625–0.25 µg/ml Ornidazole: 0.0625–0.25 µg/ml	^60^
E. h	Clinical isolates	6	Axenic/Xenic	Israel	Culture tube method	Quantitative observations with eosine stain	IC50- Metronidazole: 1–10 µg/ml	^36^
E. h (HM1: IMSS)	Standard strain	1	Axenic	Virginia, U.S	Micro dilution plate method	Quantitative observations with trypan blue exclusion	MIC- Metronidazole: 20 µg/ml	^61^
E. h	Clinical isolates	10	NA	Santo Domingo, Dominican Republic	NA	NA	Metronidazole: 25 mg/l	^62^
E. h	Clinical isolates	4	Axenic	Queensland Australia	Micro dilution plate method	Motility and rounding up	MIC- Metronidazole: 12.5 -25 µM	^31^
E. h	Standard/ clinical strains	14	NA	London, U.K	Micro dilution plate method	Radioactivity of methyl-thymidine	IC50- Metronidazole: 18.47 µM	^32^
E. h E. d	Clinical isolates	15 30	Mono- xenic	Chandigarh India	Micro dilution plate method	Nitroblue tetrazolium (NBT) reduction method	IC50 for E. h/E.d- Metronidazole: 13.2/15.6 Chloroquine: 26.3/28.9 Emetine: 31.2/32.8 Tinidazole: 12.4/13.2	^10^
E. h	Clinical isolates	1	Poly- Xenic	Cameroon, Africa	Culture tube method	Viability count using trypan blue exclusion technique	EC50- Metronidazole: 46 µg/ml	^63^
E. h (HM1: IMSS)	Standard strain	1	Axenic	Mexico	Vial-micro method	Quantitative assessment	IC50- Metronidazole: 0.711 µg/ml Tinidazole: 0.160 µg/ml Emetine 1.630 µg/ml Ornidazole 0.120 µg/ml Secnidazole: 0.161 µg/ml	^64^
E. h (MS275030)	Standard strain	1	Axenic	Dhaka, Bangladesh	24-well culture plate method	Quantitative assessment	MIC- Metronidazole: < 0.8 mg/ml	^33^
E. h (HM1: IMSS)	Standard strain	1	Axenic	Malaysia	Microdilution plate method	Motility and rounding up	MIC- Metronidazole: 6.25 µg/ml MAC- Metronidazole: 12.5 µg/ml	^65^
E. h	Clinical isolates	NA	Xenic	Tehran, Iran	NA	Mobility and tonality using 0.01% Eosin	IC- Metronidazole: 2 mg/ml	^34^
E. h	Clinical isolates	15	Mono- xenic	India	Microdilution plate method	Nitroblue tetrazolium (NBT) reduction method	IC50- Metronidazole: 20 µM Tinidazole: 16.01 µM	This Study

E. h—*E. histolytica*; E. d—*E. dispar*; NA—Not available; ED50—Effective dose 50; MIC—Minimum inhibitory concentration; IC50—Inhibitory concentration 50; EC50—Effective concentration 50; MAC—Minimum amoebicidal concentration; IC—Inhibitory concentration.

### Anti-amoebic property of active compounds of *A. paniculata*

**Figure 3:**
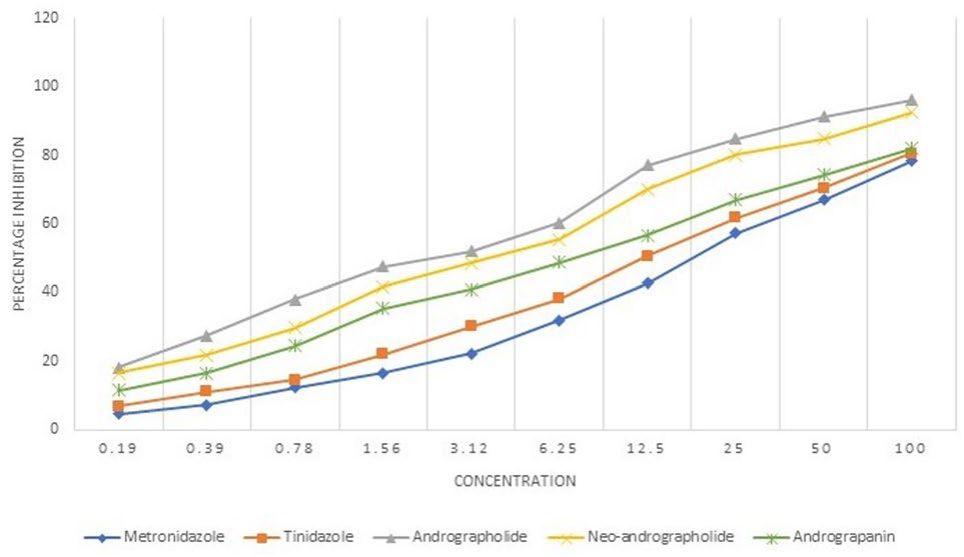
Percentage inhibition of *E. histolytica* clinical isolates by andrographolide, neo-andrographolide and andrograpanin in comparison to the metronidazole and tinidazole.

The mean IC50 value of the *E. histolytica* isolates against the active compounds and the control drug, metronidazole has been shown in Table 2. Andrographolide showed the minimum mean IC50 value (3.06 µM) against the *E. histolytica* clinical isolates. The graphical representation of the comparison of mean percentage inhibition of the 15 clinical isolates of *E. histolytica* by the active compounds and the drugs, metronidazole and tinidazole has been shown in Fig. 3. Among the active compounds andrographolide showed the maximum percentage inhibition followed by neo-andrographolide and andrograpanin. All the three active compounds showed better percentage inhibition in comparison to the commonly used nitroimidazoles. The activities of the active compounds as compared to each other was not found to be significant. However, there was a significant percentage inhibition of the *E. histolytica* isolates by andrographolide as compared to the metronidazole (*p* = 0.0495).

**Table 2:** Mean IC50 value of the active compounds and the control drug against *E. histolytica* clinical isolates.

Anti-amoebic agent	Mean IC50 value (µM)	*p* value
Andrographolide	3.06	0.0495*
Neo-andrographolide	4.85	0.107
Andrograpanin	10.5	0.3131
Metronidazole	20.01	NA

*p value significant.

*NA* not applicable.

### Prevalence of *nim* genes

None of the *E. histolytica* isolates showed the presence of *nim* gene. In the case of stool samples, the prevalence of *nim* gene in diarrheal and non-diarrheal stool samples was found to be 76.6% (69/90) and 62.2% (56/90) respectively. The prevalence of *nim* gene was significantly more in the diarrheal samples (*p* = 0.036). Among the three *E. histolytica* positive diarrheal samples 2 (66.6%) samples showed the presence of *nim* gene and out of 87 *E. histolytica* negative diarrheal samples 67 (77%) showed the presence of *nim* gene. No significant correlation between the presence of *nim* gene in the *E. histolytica* positive and negative diarrheal samples was found (*p* = 0.68).

**Figure 4:**
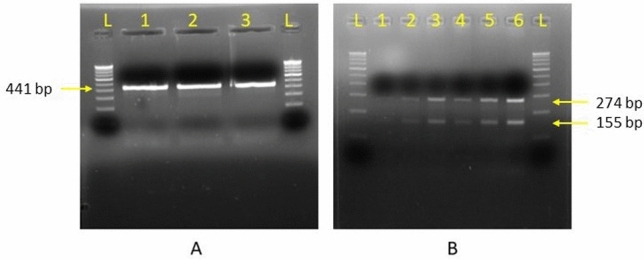
Restriction digestion products obtained by (**A**) *Hin1II* enzyme; 441 bp and (**B**) *Taq1* enzyme; 274 bp and 155 bp.

Through analysis of the digested fragments from both the enzymes, all the samples positive for *nim* gene were found to be *nim E* type. The fragments obtained by digestion from *Hin1II* and *Taq1* restriction enzyme has been shown in Fig. 4. The restriction digestion products obtained by *Hin1II* enzyme showed a single fragment of 441 bp and *Taq1* enzyme produced two fragments of 274 bp and 155 bp on agarose gel electrophoresis.

The sequence analysis of the *nim* gene PCR products confirmed the presence of *nim E* gene type. A sequence homology of 99% with the *nim E* gene type was revealed through Basic local alignment search tool (BLAST) analysis from the available database. (Accession number: AM117602.1, https://www.ncbi.nlm.nih.gov/nuccore/AM117602.1/).

### Bacterial flora in the diarrheal samples

Among the microflora associated with diarrheal samples *Prevotella* (34, 37.7%) was predominant followed by *Bacteroides* (20, 22.2%), *Escherichia coli* (16, 17.7%), *Klebsiella* (12, 13.3%) and *C. freundii* (8, 8.8%). Majority (54, 78.2%) of the diarrheal samples showing the presence of *nim* gene showed the presence of either *Prevotella* or *Bacteroides.*

## Discussion

Drug susceptibility testing in *E. histolytica* has been challenging because of the absence of competent screening assay. Additionally, the available methods are exhaustive and have complex procedures besides requiring expertise and parasite culture facilities^30^. Thus, only a handful of studies are available reporting the drug sensitivity of *E. histolytica* isolates.

The most interesting point was that though the findings of the present study were in agreement with the previous reports, yet the mean IC50 values in the present study were higher than the prior studies^10,31^. A study conducted 18 years before in India on the drug sensitivity of the *E. histolytica* isolates had shown the mean IC50 value of 13.2 µM and 12.4 µM for metronidazole and tinidazole respectively^10^. The increase in the mean IC50 values can be attributed to the emergence of clinical resistance or due to the differences in the culture techniques of the parasite, strains involved or raw materials used. Studies have reported the minimum MIC of 12.5–25 µM for the laboratory passaged *E. histolytica* strains. Another study described mean IC50 value of 18.47 µM and a cut off value of > 30 µM for resistance in the *E. histolytica* isolates^32^. However, majority of the studies on the drug sensitivity of *E. histolytica* are outdated and there is a dearth of recent data against the commonly used anti-amoebic agents. Additionally, the comparison of the available studies regarding the drug sensitivity of *E. histolytica* particularly becomes difficult because of the use of the different methods for the measurement of the activity of the drugs.

Various studies have reported the sensitivity of the drugs against the *E. histolytica* isolates in terms of minimum lethal concentration (MLC), MIC, IC50, effective dose 50 (ED50) etc^32–35^. However, it has been suggested to report the sensitivity of a drug as molar concentration (µM) to standardize the comparison of efficacy of the drugs especially in cases when metronidazole is compared with other nitroimidazoles with significantly higher molecular weight^31^. The clinical isolates included in the present study were maintained in the monoxenic culture as it has been previously reported that presence of bacterial flora along with the amoeba did not significantly interfere with the performance of the test or the sensitivity values^10,36^.

The percentage inhibition of the *E. histolytica* clinical isolates by marker active compound andrographolide was significantly higher than the control drug metronidazole. Reports have suggested that andrographolide and its analogues possess some novel mechanism of action responsible for their effects^37^. A recent study using andrographolide based nanoparticles has shown that they generate intracellular reactive oxygen species that promote damage to the vital biomolecules including DNA, proteins and lipids and cause membrane leaking leading to the release of the cytoplasmic components and death^38^.

In the malarial parasite, andrographolide had shown inhibitory activity towards the ring-stage of the parasite affecting the protein and nucleic acid synthesis. Andrographolide has been designated as the ‘Transcription blocker’ in the parasite *Plasmodium falciparum*^39^. In the andrographolide treated filarial parasite the activity of antioxidant enzymes and glutathione-S-transferase (GSH) has been found to be reduced in concentration dependent manner^40^. Additionally, in parasites treated with andrographolide an increase in the activity of NADPH oxidase has been seen which leads to the development of hydrogen peroxide (H_2_O_2_) and other hazardous reactive oxygen species splitting the mitochondrial membrane organization^40^. Even though andrographolide has been found to be effective as a traditional medicine in dysentery, a detailed study on the exact mode of action of andrographolide in *E. histolytica* is lacking.

In the present study, the *nim* gene was not detected in any of the clinical isolates of *E. histolytica*. However, the present hospital-based study revealed high level prevalence of the *nim* gene in the diarrheal as well as non-diarrheal stool samples of the patients. In other anaerobic micro-organisms, such as clinical isolates of *Bacteroides* spp. and *Prevotella* spp. the carriage rate of 0–2.8% and 0–8% respectively has been reported^17,41–43^. There is no data on the presence of *nim* genes in *E. histolytica* clinical isolates for commenting on this study finding. The *nim* gene associated metronidazole resistance has been known to vary among the diverse geographical regions^44^. In the present study the decreased susceptibility of the clinical isolates of *E. histolytica* towards commonly used nitroimidazoles does not seem to be associated with the *nim* genes*.* Previous studies have suggested that the reduction in sensitivity to metronidazole in the *E. histolytica* isolates can be because of several factors, some of which are non-enzymatic^45^. The increased expression of the iron super dismutase (Fe-SOD) and peroxiredoxin along with the decreased expression of ferredoxin1 and TrxR constitutes important component involved in the mechanism of metronidazole resistance in *E. histolytica*^46^.

High prevalence of *nim* gene in stool samples of the patients attending the tertiary care centre is in concordance with another study from India that showed high copy number of the *nim* gene in the stool samples of the patients with gastrointestinal discomfort as well as in the healthy individual from the community^47^. Such high prevalence of the *nim* gene in this geographical region can be attributed to the OTC sales of the nitroimidazoles (especially metronidazole), which has been linked with the increased frequency of the *nim* gene induction^48^. Also, the presence of *nim* gene was found to be significantly associated with the diarrheal stool samples (*p* = 0.036). This could be due to the significant abundance of the *nim* gene carrying micro-organisms such as *Bacteroides* spp. and *Prevotella* spp. in the diarrheal stool samples^49,50^.

The presence of *nim E* gene type in all the positive samples has been reported in the present study. Our finding is in-line with the previous studies reported from India which described *nim E* to be the most common *nim* gene type circulating in this geographical region^5,47,51^. In contrast to our results, studies from other part of the world have shown *nim A* gene followed by *nim B* and *nim D* gene to be most prevalent type^14,15^. However, no possible correlation between the type of *nim* gene and the degree of resistance has been ever described in the literature.

The scope of the present work was to analyse the susceptibility pattern of the clinical isolates of *E. histolytica* towards the commonly used drugs. Along with it, the anti-amoebic property of the marker compounds of *A. paniculata* was studied. To the best of our knowledge, this is the first report of the anti-amoebic activity of the active compounds from the extracts of leaves of *A. paniculata* against the clinical isolates of *E. histolytica*. A detailed study of the toxicity profile, dose determination and the delivery options of the active compounds is required to establish them as a potent anti-amoebic agent in the future.

The study was not without any limitation as only 15 clinical isolates of *E. histolytica* were included in the study owing to the difficulty in the establishment and maintenance of the parasitic culture. The drug susceptibility assays could not be carried out in the axenic culture due to lack of the culture and maintenance facility. Additionally, the drug susceptibility assay was performed using only two drugs, metronidazole and tinidazole. However, these drugs are the main line of treatment for the *E. histolytica* infections including ALA cases and therefore provides important insight into the situation.

## Material and methods

### Ethics statement

The study was ethically approved by Institute Ethical Committee, Faculty of Medicine, Institute of Medical Sciences, Banaras Hindu University (Dean/2016–17/EC/045). All experiments in the present study were performed in accordance with guidelines and regulations provided by the Institute Ethical Committee. The study included only adults and written informed consent was obtained from all the subjects who participated after explaining them the purpose of the study.

### Inclusion/exclusion criteria

Patients with well-defined liver abscesses greater than 5 cm diameter which were confirmed through abdominal ultrasound were included in the present study. Patients who have gone through aspiration previously or were on any medication from past four weeks were excluded from the study. Furthermore, the liver aspirate showing presence of associated bacteria either by aerobic culture or by molecular detection through 16-S rRNA primers were excluded from the study^52^.

### Clinical samples and isolates of *E. histolytica*

Fifteen isolates from 15 *E. histolytica* positive ALA samples from patients attending the Gastroenterology and Radiology department of Sir Sunderlal Hospital, Varanasi, India were included in this study. As the trophozoites are majorly attached to the wall of the abscess, the use of a series of smaller bottles for the collection of the liver aspirate was done to keep the last fraction undiluted by the main mass of material^53^.

The stool samples from these ALA patients were also collected. A pre-tested questionnaire about the demographic details including age, gender, residence, education, employment and economic status of the patients were collected through experienced research scholars.

Additionally, diarrheal (n = 90) and non-diarrheal (n = 90) stool samples from patients attending different outpatient departments of Sir Sundarlal Hospital, Varanasi were also included to detect the frequency of the *nim* genes in this geographical region. All the diarrheal samples were inoculated on the Mac Conkey agar medium and blood agar medium for overnight incubation at 37 °C. Additionally screening of common anaerobes from previous literature was done in the diarrheal samples using conventional PCR^29^.

### Confirmation of the samples

Direct microscopy was performed for all the samples through wet mount to screen for the presence of trophozoites of *Entamoeba* spp. Nested multiplex PCR was performed for the molecular detection of *E. histolytica* in all the samples using species specific primers targeting 16S like rRNA gene^54^. The reaction mixture preparation and reaction conditions were as described previously^29^.

### Isolation and culture of the trophozoites

Trophozoites were isolated from liver aspirate of ALA patients by culture in modified Boeck and Drbohlav’s monoxenic medium with few modifications^53,55^. Locke-egg medium was prepared. For this, Locke’s solution was prepared by dissolving the 8.0 g sodium chloride (Sigma-Aldrich Chemicals Pvt. Ltd, India), 0.2 g calcium chloride (Sigma-Aldrich Chemicals Pvt. Ltd, India), 0.2 g potassium chloride (Sigma-Aldrich Chemicals Pvt. Ltd, India), 0.01 magnesium chloride (Sigma-Aldrich Chemicals Pvt. Ltd, India), 2.0 g sodium phosphate (Sigma-Aldrich Chemicals Pvt. Ltd, India), 0.4 g sodium bicarbonate (Sigma-Aldrich Chemicals Pvt. Ltd, India) and 0.3 g potassium phosphate, monobasic (Sigma-Aldrich Chemicals Pvt. Ltd, India) into 1L distilled water. The Locke solution was autoclaved at 15 min at 121 °C under a pressure of 15 lb/in^2^. Any precipitate formed was removed by filtration by Whatman no. 1 paper.

For egg slant preparation, fresh hens’ eggs were sterilized by flaming in 70% ethanol and broken into a graduated cylinder. Locke’s solution (12.5 ml) per 45 ml of egg was added and then emulsified in a Waring-type blender and filtered through gauze into a flask. A total of 5 ml of the emulsified egg were added to standard culture tubes (16 by 125 mm) and sterilized by inspissation. After cooling of the slants, they were overlayed with 6 ml of Locke’s solution.

Deactivated bovine serum (Biological industries, Kibbutz Beit-Haemek, Israel) and sterilized rice starch (VWR International, Poole, United Kingdom) were added to the medium before addition of the samples^56^. Along with it, a loopful of pure colonies of *Klebsiella pneumoniae* which was susceptible to the metronidazole was inoculated to the culture tubes before the addition of the clinical sample. The sub-culturing was performed every 48 h and the trophozoites were further subjected to drug susceptibility testing mostly after two passages.

### Chemicals and antimicrobial agents

The pure salts of drug, metronidazole and tinidazole were included in the study (Sigma-Aldrich Chemicals Pvt. Ltd, India) The concentration range of 0.78 µM—100 µM were used. Andrographolide (CAS No. 5508–58–7, purity > 95%), neoandrographolide (CAS.No. 27215–14–1, purity > 95%) and andrograpanin (CAS No. 82209–74–3, purity > 95%) were obtained from Natural Remedies Pvt. Ltd (Bangalore, India) and used in the concentration of 0.19 µM—100 µM.

### Drug susceptibility testing (DST)

For the susceptibility testing, Robinson medium for *Entamoeba* twin pack (HiMedia laboratories Pvt. Ltd, Mumbai, India) was used. Susceptibility testing to the standard drugs and the active compounds was performed as described previously^10^. Briefly, the trophozoites were harvested from 24 h old culture from the interface of the locke’s solution and the egg slant and the count were adjusted to 1 × 10^5^ trophozoites per ml. The *in-vitro* susceptibility testing was performed in 96 wells microtiter plates. The doubling dilutions of the drugs and active compounds was performed to get the required concentrations. Diluted trophozoite suspension was added and the plates were incubated for 4 h at 37 °C. Following incubation, the plate’s content was discarded and the plate was washed. Further, 100 µl of nitroblue tetrazolium (HiMedia laboratories Pvt. Ltd, Mumbai, India) was added to each well and the plates were incubated at 37 °C for 45 min. After incubation, the plates were again washed and DMSO (200 µl/well) was added. Thereafter the plates were incubated at 37 °C for 10 min. Following incubation, the optical density (OD) was measured. Each isolate was tested against the drugs and active compounds in duplicates. Each test included a control well containing only media and the trophozoites without drugs and a blank well containing only media. The measurement of OD in each test was done at 540 nm using microtiter plate reader (LisaScan® EM Elisa plate reader, Transasia Bio-Medicals Ltd, India). The percentage of non-viable trophozoites at each concentration was calculated using the formula:

$${\text{Percentage of non-viable trophozoites}} = \frac{{{\text{Test OD} - \text{Blank OD}}}}{{{\text{Control OD} - \text{Blank OD}}}}{\text{ }} \times {\text{ 1}}00$$

The mean IC_50_ values for all the clinical isolates against the drugs (metronidazole, tinidazole) and the active compounds (andrographolide, neoandrographolide and andrograpanin) was calculated using "Quest Graph™ IC50 Calculator” (AAT Bioquest, Inc.)

### Screening of the *nim* gene

The 15 *E. histolytica* isolates and stool samples from the diarrheal and non-diarrheal patients were screened for the presence of *nim* genes. DNA extraction was carried out from all the isolates and the stool samples by using QIAGEN stool mini kit (Qiagen, Germany) as per the manufacturer’s instructions.

The detection of *nim* genes in the *E. histolytica* isolates and stool samples was carried out by conventional PCR using the universal set of primers, Nim-3 and Nim-5, for all known *nim* genes^57^.

### Determination of *nim* gene type

The determintion of the *nim* gene type was done by restriction fragment length polymorphism (RFLP). The PCR products obtained in the previous step were digested by using two restriction enzymes *Taq1* (New England Biolabs, Massachusetts, United States) and *Hin1II* (New England Biolabs, Massachusetts, United States) as per the manufacturer’s instructions^15,58^. For *nim* gene type confirmation, PCR products were subjected to DNA sequencing of the partial region of SSU rRNA gene.

### Statistical analysis

The mean IC50 value of the clinical isolates of *E. histolytica* were compared by Student’s t-test using 2019 MedCalc software (version:bv). The presence of the *nim* gene in diarrheal and non-diarrheal stool samples was also compared using 2019 MedCalc software (version:bv). In the case of the active compounds, metronidazole was used as the control drug. A *p *value < 0.05 was considered to be significant.

## Data Availability

All the data generated or analysed during the study has been included in the manuscript.
